# Accumbens Cholinergic Interneurons Mediate Cue-Induced Nicotine Seeking and Associated Glutamatergic Plasticity

**DOI:** 10.1523/ENEURO.0276-20.2020

**Published:** 2021-02-02

**Authors:** Jonna M. Leyrer-Jackson, Michael Holter, Paula F. Overby, Jason M. Newbern, Michael D. Scofield, M. Foster Olive, Cassandra D. Gipson

**Affiliations:** 1 Department of Psychology, Arizona State University, Tempe, AZ 85281; 2 Department of Neuroscience, School of Life Sciences, Arizona State University, Tempe, AZ 85281; 3 Department of Anesthesiology, Medical University of South Carolina, Charleston, SC 29425; 4 Department of Family and Community Medicine, University of Kentucky, Lexington, KY 40536

**Keywords:** accumbens, cholinergic, glutamate, nicotine, plasticity, relapse

## Abstract

Nicotine, the primary addictive substance in tobacco, is widely abused. Relapse to cues associated with nicotine results in increased glutamate release within nucleus accumbens core (NAcore), modifying synaptic plasticity of medium spiny neurons (MSNs), which contributes to reinstatement of nicotine seeking. However, the role of cholinergic interneurons (ChIs) within the NAcore in mediating these neurobehavioral processes is unknown. ChIs represent less than 1% of the accumbens neuronal population and are activated during drug seeking and reward-predicting events. Thus, we hypothesized that ChIs may play a significant role in mediating glutamatergic plasticity that underlies nicotine-seeking behavior. Using chemogenetics in transgenic rats expressing Cre under the control of the choline acetyltransferase (ChAT) promoter, ChIs were bidirectionally manipulated before cue-induced reinstatement. Following nicotine self-administration and extinction, ChIs were activated or inhibited before a cue reinstatement session. Following reinstatement, whole-cell electrophysiology from NAcore MSNs was used to assess changes in plasticity, measured via AMPA/NMDA (A/N) ratios. Chemogenetic inhibition of ChIs inhibited cued nicotine seeking and resulted in decreased A/N, relative to control animals, whereas activation of ChIs was unaltered, demonstrating that ChI inhibition may modulate plasticity underlying cue-induced nicotine seeking. These results demonstrate that ChI neurons play an important role in mediating cue-induced nicotine reinstatement and underlying synaptic plasticity within the NAcore.

## Significance Statement

The studies reported here are the first to address the role of cholinergic interneurons (ChIs) in cue-induced nicotine seeking and in nicotine-induced changes in synaptic plasticity within nucleus accumbens medium spiny neurons (MSNs). Chemogenetic inhibition of ChIs prevented cue-induced nicotine seeking and associated MSN plasticity. Additionally, these studies highlight the role of nicotinic acetylcholine receptors (nAChRs) in mediating cue-induced nicotine seeking and associated MSN morphology. Through the use of chemogenetics, behavioral assessments, and electrophysiology the results presented here highlight the importance of the cholinergic circuitry within the nucleus accumbens core (NAcore) for mediating cue-induced nicotine seeking.

## Introduction

Tobacco use disorder is the leading preventable cause of death within the United States and represents a substantial burden to public health (Prochaska and Benowitz, 2016). Self-administration of nicotine produces robust cellular adaptations within brain regions associated with drug reward, including the nucleus accumbens core (NAcore; Dani et al., 2001; Mansvelder et al., 2009; Gipson et al., 2013b; Scofield et al., 2016). The effects of nicotine on dopaminergic neurons within the ventral tegmental area (VTA) are perhaps the most prominently characterized synaptic alterations. Specifically, nicotine has been shown to increase glutamatergic input onto dopaminergic neurons, as measured by changes in AMPA/NMDA (A/N) ratios, and has been found to potentiate GABAergic input onto local VTA inhibitory neurons. Together, these findings support that nicotine exposure enhances VTA dopaminergic excitability and dopamine release into target areas, including the nucleus accumbens (Mansvelder and McGehee, 2000; Brown et al., 2010; Grieder et al., 2012). Enhanced dopaminergic release within the accumbens is hypothesized to potentiate medium spiny neuron (MSN) synapses as well as their response to glutamatergic stimulation (Pistillo et al., 2015). In support, increases in MSN spine length and density are observed in rats chronically exposed to nicotine (Brown and Kolb, 2001), an effect associated with enhanced NMDA receptor mediated currents within the NAcore. Further, drugs of abuse including nicotine induce changes in glutamatergic homeostasis within the mesocorticolimbic brain circuitry (Kalivas, 2009), thus altering glutamatergic-MSN connectivity and associated synaptic plasticity within the accumbens. In fact, cue-induced nicotine seeking has been shown to alter glutamatergic plasticity within the NAcore, enhancing the A/N of MSNs in a rapid and transient manner as well as morphologic increases in dendritic spine head diameter (Gipson et al., 2013b). Thus, while prior studies have explored changes in MSN synaptic plasticity because of altered dopaminergic and glutamatergic signaling, current studies have yet to explore additional mechanisms driving changes in MSN synaptic plasticity, which may underlie nicotine-seeking behaviors.

Cholinergic interneurons (ChIs) account for <1% of the cell population within the accumbens, yet they have the ability to exert powerful modulatory control over accumbens circuitry (Zhou et al., 2002; Tepper and Bolam, 2004) and may play a role in synaptic plasticity of MSNs. ChIs provide most of the intrinsic cholinergic innervation of the NAcore and are widely distributed throughout the striatum (Girasole and Nelson, 2015). Additionally, ChIs provide cholinergic modulation of striatal dopaminergic transmission and are known to co-release ACh and glutamate (Zhou et al., 2001; Cachope et al., 2012; Threlfell et al., 2012; Kljakic et al., 2017), allowing for additional modulation of MSN activity (Witten et al., 2010; Oldenburg and Ding, 2011). Importantly, NAcore ChIs are involved in reward-predicting events (Atallah et al., 2014) and extracellular ACh is elevated following drug intake (Rada et al., 2001; Mansvelder et al., 2003; Crespo et al., 2006; Yee et al., 2011). In fact, acquisition of cocaine and remifentanil is paralleled with an increase in ACh release. Specifically, an overflow of ACh was observed following drug delivery, and blocking nicotinic acetylcholine receptors (nAChRs) was found to inhibit drug acquisition (Crespo et al., 2006). Together, these studies indicate that ChI-induced activation of nAChRs plays an important role in driving motivated drug use.

Increased glutamate release from prelimbic afferents targeting the NAcore increases cue-induced drug seeking (Stefanik et al., 2016) by modulating MSN synaptic physiology (Gipson et al., 2013b; Quintero, 2013; Alasmari et al., 2016). Given that NAcore ChIs are involved in drug-motivated behavior, we hypothesized that this small population of cells mediates glutamatergic plasticity and nicotine-seeking behavior. Using choline acetyltransferase (ChAT) cre-recombinase transgenic rats (ChAT::cre), ChI activity was bidirectionally manipulated via viral administration of cre-dependent designer receptors exclusively activated by designer drugs (DREADDS) before cue-induced reinstatement to examine the modulatory role of ChIs over nicotine-seeking behaviors. Whole-cell electrophysiological recordings were then conducted from NAcore MSNs to determine whether modulation of ChI activity mediates MSN synaptic plasticity measured via A/N currents.

## Materials and Methods

### Subjects

Sixty- to 90-d-old adult Long–Evans male (*N* = 17) and female (*N* = 20) rats were housed in a temperature and humidity-controlled animal facility on a 12/12 h dark/light reverse cycle and had *ad libitum* access to food and water before experimentation. All procedures were approved by the Institutional Animal Care and Use Committee (IACUC) of Arizona State University. All animals used for chemogenetic ChI manipulation were bred in-house and confirmed ChAT::cre-positive through genotyping described below. Breeder ChAT::cre-positive males (Long–Evans- Tg(ChAT-cre5)5.1 Deis) were purchased from Rat Resource and Research Center (RRRC, RRC#658) and bred in-house with Long–Evans wild-type females purchased from Envigo.

All ChAT::cre self-administration data presented here are included within a larger data set exploring the role of sex and strain on nicotine self-administration in our recently published study (Leyrer-Jackson et al., 2020). However, while these animals were included within the Long–Evans male and female groups, the study did not focus on the subset of animals as reported here, but rather included in a larger dataset focusing on self-administration parameters across strain and sex.

### ChAT::cre genotyping

Tail snips were collected from all animals at PND 10. Subjects were genotyped using the following primers: 5′-AGA GTA CAC TGT GGG CAG GA-3′ (R658.F2 located within the promotor region of ChAT; forward primer) and 5′-GCA AAC GGA CAG AAG CAT TT-3′ (Cre.R located in cre-recombinase reverse primer). Using standard PCR-based genotyping, all animals used were confirmed ChAT::cre transgene-positive.

### Surgical procedures

All rats were anesthetized using ketamine hydrochloride (80–100 mg/kg, i.m.) and xylazine (8 mg/kg, i.m.) and underwent surgical implantation of intravenous jugular catheters as well as stereotaxically implanted guide cannulae targeting the NAcore as previously described (Leyrer-Jackson et al., 2020; Namba et al., 2020). Intravenous jugular catheters (made from polyurethane tubing; BTPU-040; Instech) were inserted 2.5–3 cm into the right jugular vein and were threaded subcutaneously to the posterior side of the animal where it was connected to an indwelling back port (Instech). Dental cement (SNAP or Ortho-Jet) was used to adhere the catheter to the port. The indwelling port was sutured using 4–0 vicryl braided suture (Ethicons) subcutaneously ∼2 cm caudal from the shoulder blades. Following jugular vein catheterization, animals were immediately transferred to a rat stereotaxic frame and NAcore guide cannulae were bilaterally aimed at the NAcore according to a stereotaxic atlas (Paxinos and Franklin, 2001). Guide cannulae were bilaterally implanted (+1.5 mm anterior/posterior, ±2.0 mm medial/lateral, and −5.5 mm dorsal/ventral; Paxinos and Franklin, 2001). Guide cannulae were positioned 2 mm dorsal to the NAcore to prevent damage to the area. Three screws were placed into the skull, where they were used as anchors (one anterior to bregma, and two posterior) for adhering dental cement to the skull to hold guide cannulae in position. Microinjectors protruded 2 mm past the guide cannulae into the NAcore. Immediately following implantation, viral vectors encoding DREADDs for ChI manipulation were infused into the NAcore of ChAT::cre animals: AAV5-hsyn-DIO-HM4D(G_i_)-mCherry (inhibitory; titer: 1.2 × 1013 vg/ml; *N* = 15; Addgene, #44362), AAV5-hsyn-DIO-rM3D(G_s_)-mCherry (excitatory; titer: 1.3 × 1013 vg/ml; *N* = 10; Addgene, #50485; packaged into an AAV5 vector by Penn Vector Core), or AAV5-hSyn-DIO-mCherry (control; i.e., only mCherry-expressing; titer: 1.5 × 1013 vg/ml; *N* = 12; Addgene, #44362) at a volume of 0.5 μl per hemisphere. Rats were immediately administered cefazolin (100 mg/kg, i.v.) and heparin (10 U/ml, i.v.) and for seven consecutive days during the recovery period. Meloxicam (1 mg/kg, s.c.) was given immediately and for the first 3 d of the recovery period. Heparin (10 U/ml, i.v.) was administered daily.

### Food training procedures

All rats underwent food training on the sixth day of postoperative care. Food restriction (20 g of chow/d) was implemented a minimum of 2 h before food training initiation and was maintained for the duration of the experiment. Food training sessions were 15 h duration, and one active lever press resulted in the delivery of one food pellet [fixed-ratio-1 (FR1), schedule of reinforcement; Bio-Serv, 45 mg/pellet) Concurrently, one food pellet was delivered every 20 min regardless of response. Light and tone stimuli were not paired with pellet administration. Food training criteria was set to 2:1 active to inactive lever presses throughout the session and a minimum of 200 active lever presses. All food training, self-administration, extinction, and reinstatement sessions were conducted in modular operant conditioning chambers (13 ENV-008, 15 ENV-007; Med Associates), which have been previously described in detail (Overby et al., 2018; Leyrer-Jackson et al., 2020).

### Intravenous nicotine self-administration, extinction, and reinstatement

Nicotine infusions (0.02 mg/kg/infusion) were paired with a compound stimulus (light+tone), and was followed by a 20-s timeout period. Nicotine was delivered across a 5.9-s duration, at a total volume of 0.1 ml of nicotine was administered per infusion. Infusions were paired with cue lights, located above each lever, and a 2900-Hz tone was presented throughout the duration of the nicotine infusion. Active lever presses during the timeout period were recorded, but did not result in additional nicotine infusions. An inactive lever was extended at all times and responses were recorded; however, presses yielded no programmed consequences or rewards. Session duration was 2 h in length and a FR1 schedule of reinforcement was used. During the first two sessions of self-administration, nicotine infusions were capped at 25 to prevent aversive effects that can accompany high nicotine intake. All animals were required to complete a minimum of 10 sessions before moving into extinction with the following criteria: ≥10 nicotine infusions obtained and ≥2:1 active/inactive lever press ratio. Additionally, extinction can be defined in two ways: (1) either by the lack of delivery of a reinforcer that was previously delivered after a response (e.g., responses on the active lever no longer result in nicotine infusions), or (2) as the absence of a contingency between response and reinforcer (e.g., the nicotine infusion occurs regardless of an animals’ response; see Rescorla and Skucy, 1969; Lattal and Lattal, 2012). Given that the reinforcer here (the nicotine infusion) is not delivered following a time-out active lever press, the latter definition applies to these types of responses and are therefore distinct from active lever presses that directly result in the initiation of a nicotine infusion. Thus, these two types of lever presses were analyzed separately in the current study.

Following 10 non-consecutive criteria making sessions, animals were moved to the extinction phase which took place in the same boxes as self-administration. During the extinction phase, active lever presses no longer resulted in nicotine infusions or associated cues. A minimum of 14 2-h extinction sessions was required for each animal. Extinction criteria were set at <30 active lever presses on the last day of extinction. For reinstatement sessions, previously paired nicotine cues were presented on active lever pressing, however no nicotine infusions were delivered. Reinstatement sessions were 15 min in duration. Animals were then immediately killed for whole-cell electrophysiological recordings. Eight animals were removed from the current study because of not meeting self-administration criteria (*N* = 5) or lack of DREADD expression/improper cannula placement (*N* = 3).

### Intra-NAcore microinjections

**Figure 1.**
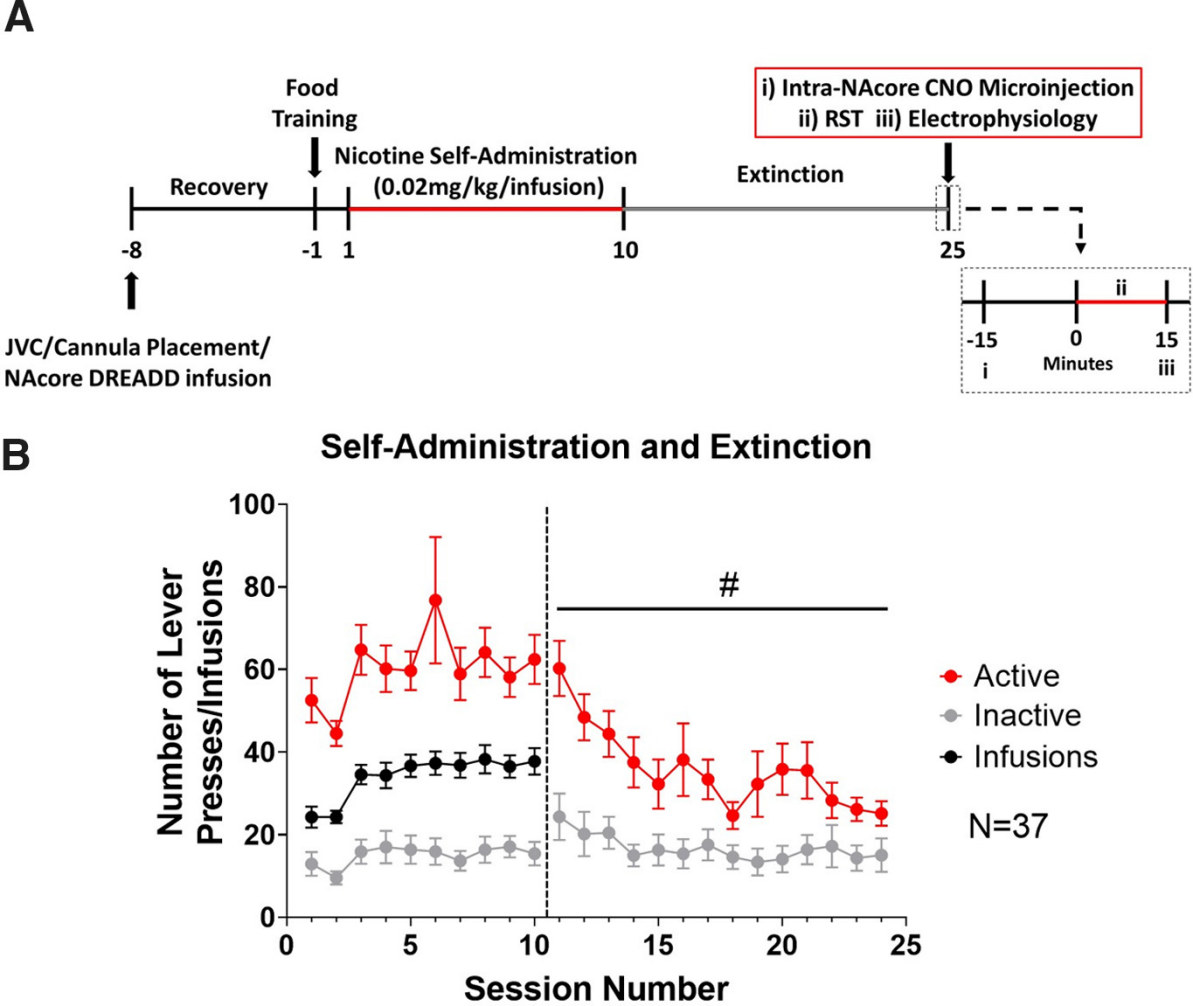
Nicotine self-administration and extinction. ***A***, A timeline of experimental procedures. JVC, jugular vein catheterization; RST, reinstatement. ***B***, Rats acquired nicotine self-administration, distinguishing between active (red) and inactive (gray) levers to receive intravenous infusions of nicotine (black). Active lever pressing was reduced across extinction sessions; #*p *<* *0.05 represents a main effect of session on active lever pressing. The vertical dotted line in ***B*** separates self-administration sessions and extinction sessions.

For ChI manipulation, bilateral intra-NAcore microinjections of clozapine N-oxide [CNO; 0.1 mg/ml dissolved in artificial CSF (aCSF)] at a volume of 0.5 μl/hemisphere were conducted 15 min before reinstatement testing (Fig. 1*A*). Intra-NAcore microinjections were chosen to avoid potential indirect effects of systemic CNO, since intracranial CNO administration does not exhibit DREADD-independent effects in multiple brain regions (for review, see Mahler and Aston-Jones, 2018).

### Electrophysiology

Following reinstatement, animals were anesthetized with CO_2_ and rapidly decapitated. Brains were rapidly removed and submerged in ice-cold carbogen (95% O_2_/5% CO_2_) saturated cutting solution (cutting aCSF) containing the following: 120 mmol/l NaCl, 25 mmol/l NaHCO_3_, 10 mmol/l dextrose, 3.3 mmol/l KCl, 1.23 mmol/l NaH_2_PO_4_, 1.8 mmol/l CaCl_2_, and 2.4 mmol/l MgCl_2_. Solution osmolarity was adjusted to 295 ± 5 mOsm and pH adjusted to 7.40 ± 0.03. Brains were then transferred to a cutting chamber of a vibrating tissue slicer (Leica, VT1000S) and 300-μm-thick coronal slices of the NAcore were prepared in ice-cold cutting aCSF. Slices were then placed in a holding chamber filled with recording aCSF solution containing the following: 120 mmol/l NaCl, 25 mmol/l NaHCO_3_, 3.3 mmol/l KCl, 1.23 mmol/l NaH_2_PO_4_, 0.9 mmol/l CaCl_2_, 2.0 mmol/l MgCl_2_, and 10 mmol/l dextrose, osmolarity adjusted to 295 ± 5 mOsm and pH adjusted to 7.40 ± 0.03. During brain slicing, cannulae placement was confirmed based on cannula track marks targeting the NAcore. In the holding chamber, aCSF was continuously bubbled with carbogen (95% O_2_/5% CO_2_) and incubated at 34°C for 45 min and then allowed to cool to room temperature before slice recording. Before experiments, slices were transferred to a recording chamber where they were perfused continuously at a flow rate of 1–2 ml/min with filtered, carbogen-saturated recording aCSF solution. MSNs were visually identified using infrared DIC microscopy with an Olympus BX51WI microscope. Additionally, a green collimated LED (ThorLabs) was used to visualize mCherry-DREADD expression within the NAcore to ensure proper expression and location before recordings. Whole-cell recordings were made from the soma of MSNs neurons after establishing a giga-ohm seal (resistance range: 1–10 GΩ). Recording pipettes (7–15 mΩ), made from thin-walled capillary tubes were filled with an intracellular solution containing the following: 135 mmol/l K-gluconate, 12 mmol/l NaCl, 1 mmol/l K-EGTA, 10 mmol/l HEPES, 2 mmol/l Mg-ATP, and 0.38 mmol/l tris-GTP. Osmolarity was adjusted to 285 ± 5 mOsm and pH adjusted to 7.30 ± 0.01. Upon establishing a giga-seal, the cell membrane was ruptured and held at –80 mV. Resting membrane potential, cellular capacitance, membrane resistance and pipet resistance were monitored throughout the duration of the recording. Only cells that exhibited thin, over shooting action potentials, normal resting membrane potential, and changes in uncompensated access resistance <20 mΩ were included in analysis. Recordings were initiated 10 min after cell membrane rupture to allow for diffusion of the internal solution into the cell. A stimulating electrode was placed in the dorsal region of the NAcore to active prelimbic excitatory fibers targeting the NAcore. AMPA currents, evoked by electrical stimulation, were first measured at −80 mV. The membrane potential was then gradually increased to +40 mV. The cell was left to stabilize at +40 mV for 5 min. EPSCs composed of both AMPA and NMDA receptor mediated currents were then elicited at +40 mV. DNQX (20 μm) was then bath applied for 5 min and NMDA receptor mediated currents were obtained. AMPA currents were then obtained by subtracting the isolated NMDA receptor-mediated current from the whole EPSC. A/N ratios were calculated by measuring the peak amplitude of each current and taking a ratio. For DREADD function validation, see procedures outlined in (Tomek et al., 2020). All recordings were conducted using the recording software Axograph. Responses were digitized at 10 kHz and saved on a disk using digidata interface (Molecular Devices) and analyzed offline using Axograph.

### Immunohistochemistry

Slices from electrophysiology were postfixed in 4% paraformaldehyde (PFA) solution for a minimum of 2 d. Slices were washed three times for 10 min each with PBS and placed into blocking solution containing PBST (PBS containing 0.1% Triton X-100) and 5% normal donkey serum. Slices were blocked for 2 h at room temperature. The mouse monoclonal Anti-ChAT antibody (1:1000; Atlas Antibodies; AMAB91130) was added to the blocking solution at a concentration of 1:1000. Tissue was incubated overnight at 4°C with gentle rocking. Slices were washed three times for 10 min each using PBST. Following the third wash, slices were incubated with Alexa Fluor 488-conjugated donkey anti-mouse IgG secondary antibody (1:200; Abcam; ab205718) in PBST (1:200) for 2 h. Slices were washed for three times using PBST under gentle agitation. Brains were mounted onto microscope slides using ProLong Gold Antifade mounting medium (ThermoFisher Scientific) and covered with cover glasses, and sealed with clear nail polish. Images were collected on a Zeiss LSM800 confocal microscope from three different tissue sections per rat with a minimum of three biological replicates. Optical sections were taken from a range of 5–15 μm from the surface of the brain tissue. All images were acquired using the same acquisition parameters, including laser power, gain, and offset.

### Drugs and viral vectors

(-)Nicotine tartrate (MP Biomedicals) was dissolved in 0.9% sterile saline and adjusted to pH 7.2–7.4 with 1 m NaOH. The final stock concentration was 0.2 mg/ml free base, which was adjusted for body weight to achieve an infusion concentration of 0.02 mg/kg/ml. CNO was purchased from Sigma-Aldrich and diluted in ACSF purchased from Tocris Biotech to 0.1 mg/ml. Heparin, xylazine (100 mg/ml), cefazolin and meloxicam used at 10 U/ml, 8 mg/kg/ml, 100 mg/kg/ml, and 1 mg/kg in 0.9% sterile saline, respectively.

### Data analysis

Analysis of self-administration, extinction and reinstatement data were performed using two-way, mixed measures ANOVA with DREADD virus as a main factor and session (extinction vs reinstatement, where applicable) as a repeated-measure factor. Electrophysiological data were analyzed using a one-way ANOVA, where DREADD virus was considered a factor. Bonferroni-corrected *t* tests *post hoc*, were conducted where appropriate. The effects of sex were examined in a separate one-way ANOVA to ensure no differences between groups before collapsing for analyses. Analysis of behavioral data only included animals that met self-administration criteria, had proper cannula placement, and expressed DREADDs within the NAcore. Linear regression analyses were used to explore the relationship between A/N and number of active lever presses. Statistical tests were performed in GraphPad Prism 8.0, and *p *<* *0.05 was considered statistically significant. Values presented are represented as mean ± SEM. All results and statistical tests ran are listed within Table 1.

**Table 1:** Statistical analyses in **Figures 1-6**

Figure location	Behavioral test	Statistical test used	Variables/comparisons	Degree of freedom	Test value	*p* value
1*B*	Self-administration	Two-way ANOVA	Session	780	1.8	0.07
			Lever	780	349.3	**<0.0001**
		Interaction	780	0.8	0.60	
	Extinction	Two-way ANOVA	Session	1092	3.2	**<0.001**
			Lever	1092	103.7	**0.0001**
		Interaction	1092	1.1	0.33	
		Bonferroni’s *post hoc*	Active vs inactive lever: session 1	1092	5.1	**<0.0001**
		Bonferroni’s *post hoc*	Active vs inactive lever: session 2	1092	4.0	**0.0009**
		Bonferroni’s *post hoc*	Active vs inactive lever: session 3	1092	3.4	**0.0096**
		Bonferroni’s *post hoc*	Active vs inactive lever: session 4	1092	3.2	**0.019**
		Bonferroni’s *post hoc*	Active vs inactive lever: session 5	1092	2.7	0.28
		Bonferroni’s *post hoc*	Active vs inactive lever: session 6	1092	3.2	**0.02**
		Bonferroni’s *post hoc*	Active vs inactive lever: session 7	1092	2.4	0.29
		Bonferroni’s *post hoc*	Active vs inactive lever: session 8	1092	1.4	0.90
		Bonferroni’s *post hoc*	Active vs inactive lever: session 9	1092	2.7	0.10
		Bonferroni’s *post hoc*	Active vs inactive lever: session 10	1092	3.1	**0.03**
		Bonferroni’s *post hoc*	Active vs inactive lever: session 10	1092	2.7	0.09
		Bonferroni’s *post hoc*	Active vs inactive lever: session 10	1092	1.6	0.82
		Bonferroni’s *post hoc*	Active vs inactive lever: session 10	1092	1.7	0.75
		Bonferroni’s *post hoc*	Active vs inactive lever: session 10	1092	1.4	0.90
None	Self-administration (reinforced vs non-reinforced)	Two-way ANOVA	Session	740	1.74	0.08
			Group (reinforced, non-reinforced)	740	13.6	**0.0002**
			Interaction	740	1.16	0.32
None	Self-administration (reinforced vs non-reinforced)	Bonferroni’s *post hoc*	Reinforced vs non-reinforced, all sessions	740	0.44–2.22	0.27 to >0.999
2*A*	Self-administration: sex differences, active lever pressing	Two-way ANOVA	Sex (male, female)	360	1.64	0.20
			Session	360	1.30	0.24
			Interaction	360	0.33	0.97
2*B*	Self-administration: sex differences, inactive lever pressing	Two-way ANOVA	Sex (male, female)	360	0.63	0.77
			Session	360	8.57	**0.004**
			Interaction	360	0.21	0.99
2*B*	Self-administration: sex differences, inactive lever pressing	Bonferroni’s *post hoc*	Male vs female all sessions	360	0.37–1.55	>0.999
2*C*	Self-administration: sex differences, infusions	Two-way ANOVA	Sex (male, female)	360	0.34	0.56
			Session	360	3.47	**0.004**
			Interaction	360	0.31	0.97
2*C*	Self-administration: sex differences, infusions	Bonferroni’s *post hoc*	Male vs female: all sessions	360	0.03–1.08	>0.999
2*D*	Self-administration: sex differences, total number of infusions	Unpaired Student’s *t* test	Sex (male, female)	36	0.25	0.80
2*E*	Extinction: sex differences, active lever pressing	Two-way ANOVA	Sex (male, female)	504	2.55	0.11
			Session	504	2.28	**0.006**
			Interaction	504	0.75	0.71
2*E*	Extinction: sex differences, active lever pressing	Bonferroni’s *post hoc*	Male vs female: all sessions	504	0.02–1.67	>0.999
2*F*	Extinction: sex differences, inactive lever pressing	Two-way ANOVA	Sex (male, female)	504	5.46	**0.02**
			Session	504	0.70	0.76
			Interaction	504	0.48	0.93
2*F*	Extinction: sex differences, inactive lever pressing	Bonferroni’s *post hoc*	Male vs female: all sessions	504	0.0007–1.7	0.74 to >0.999
3*A*	Self-administration: DREADD treatment, active lever pressing	Two-way ANOVA	Treatment (G_s_, G_i_, control)	350	8.72	**0.0002**
			Session	350	1.21	0.29
			Interaction	350	0.56	0.92
3*B*	Self-administration: DREADD treatment, inactive lever pressing	Two-way ANOVA	Treatment (G_s_, G_i_, control)	350	6.65	**0.002**
			Session	350	1.04	0.411
			Interaction	350	0.65	0.86
3*C*	Self-administration: DREADD treatment, infusions	Two-way ANOVA	Treatment (G_s_, G_i_, control)	350	0.57	0.56
			Session	350	3.18	**0.001**
			Interaction	350	0.33	0.99
3*D*	Self-administration: DREADD treatment, total number of infusions	One-way ANOVA	Treatment (G_s_, G_i_, control)	35	1.25	0.90
3*E*	Extinction: DREADD treatment, active lever pressing	Two-way ANOVA	Treatment (G_s_, G_i_, control)	504	12.74	**<0.0001**
			Session	504	2.21	**0.008**
			Interaction	504	0.80	0.75
3*F*	Extinction: DREADD treatment, inactive lever pressing	Two-way ANOVA	Treatment (G_s_, G_i_, control)	504	10.52	**<0.0001**
			Session	504	0.67	0.79
			Interaction	504	0.21	>0.99
4*C*	Reinstatement	Two-way ANOVA	Treatment (G_s_, G_i_, control)	70	8.43	**0.0005**
			Session	70	13.24	**0.0005**
			Interaction	70	6.84	**0.0019**
4*C*		Bonferroni’s *post hoc*	Control extinction vs control reinstatement	70	4.43	**0.0005**
4*C*		Bonferroni’s *post hoc*	G_s_ extinction vs G_s_ reinstatement	70	2.2	**0.03**
4*C*		Bonferroni’s *post hoc*	G_i_ extinction vs G_i_ reinstatement	70	0.55	0.999
4*D*	A/N ratio	Ordinary one-way ANOVA	Treatment (G_s_, G_i_, control)	25	5.7	**0.009**
4*D*		Bonferroni’s *post hoc*	Control vs inhibitory	25	3.3	**0.009**
5*A*	Membrane capacitance	Ordinary one-way ANOVA	Treatment (G_s_, G_i_, control)	25	0.38	0.69
5*B*	Resting membrane potential	Ordinary one-way ANOVA	Treatment (G_s_, G_i_, control)	25	3.3	0.054
5*C*	NMDA decay	Ordinary one-way ANOVA	Treatment (G_s_, G_i_, control)	25	5.7	**0.009**
6	Reinstatement active lever pressing correlation to A/N	Linear regression analysis	Active lever pressing vs A/N ratio	26	12.37	**0.002**

Bolded values indicate statistical significance.

## Results

### Nicotine self-administration and extinction

A two-way ANOVA revealed that active lever pressing remained higher than inactive lever pressing across self-administration sessions (*F*_(9,780)_ = 349.3; *p *<* *0.05; Fig. 1*B*). No interaction between session and lever was observed (*p *>* *0.05; Fig. 1*B*). We next compared reinforced and non-reinforced active lever pressing. A two-way ANOVA where session and group (reinforced and non-reinforced active lever pressing) were considered main effects revealed an effect of group (*F*_(1,740)_ = 13.6; *p *<* *0.05) but not session (*p *>* *0.05) and no interaction was observed (*p* < 0.05). However, a Bonferroni’s *post hoc* comparison showed no differences between reinforced and non-reinforced lever pressing across session (*p *>* *0.05). For extinction, a two-way ANOVA revealed that active lever pressing decreased across sessions (*F*_(13,1092)_ = 3.2; *p *<* *0.05; Fig. 1*B*). Further, a *post hoc* comparison revealed that at the beginning of extinction, active lever pressing was higher than inactive lever pressing (*t*_(1092)_ = 5.1; *p *<* *0.05; Fig. 1*B*). However, for the last four sessions, active and inactive lever pressing were not statistically different (*p *>* *0.05; Fig. 1*B*).

#### Sex differences in self-administration and extinction

**Figure 2.**
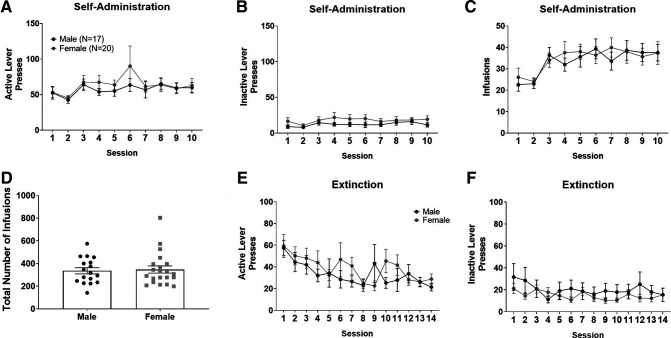
Male and female rats did not differ in nicotine self-administration or extinction of lever pressing. No differences were found between male and female rats in active (***A***) or inactive (***B***) lever pressing throughout self-administration. Additionally, they did not differ in the number of infusions across self-administration sessions (***C***) or the total number of nicotine infusions earned throughout the 10 non-consecutive criteria-making sessions (***D***). Data points within ***D*** represent individual animal values. Males and females did not differ in active (***E***) or inactive (***F***) lever pressing throughout extinction. Male and female rats are depicted in black and gray, respectively. Numbers in legend of panel ***A*** represent the number of animals in each group.

We next examined differences between males and females throughout self-administration and extinction using a two-way ANOVA, where session and sex (male and female) were considered factors. No differences in active lever pressing between sexes across self-administration sessions were observed (*p *>* *0.05; Fig. 2). An effect of session on inactive lever pressing (*F*_(1,360)_ = 8.6; *p* < 0.01) and infusions (*F*_(1,360)_ = 3.5; *p* < 0.01) was observed. However, *post hoc* analysis revealed no within session differences between males and females for inactive lever pressing (*p* > 0.05) or infusions (*p *>* *0.05), suggesting that females and males do not differ within sessions.

During extinction, an effect of session on active lever pressing was observed throughout sessions (*F*_(13,504)_ = 2.28; *p* < 0.01). A *post hoc* comparison revealed no differences between sexes within sessions (*p* > 0.05). Additionally, an effect of sex on inactive lever pressing was observed throughout extinction sessions (*F*_(13,504)_ = 5.46; *p* < 0.05), yet a *post hoc* comparison revealed no differences within sessions. Additionally, no differences in the total number of infusions earned across the 10-criteria making sessions were observed between male (*N* = 17) and female (*N* = 20) animals (*p *>* *0.05; Fig. 2). These results are consistent with our previous study, where we found no differences in total infusions or number of infusions across self-administration sessions between Long–Evans male and female rats (Leyrer-Jackson et al., 2020).

#### DREADD-type differences in self-administration and extinction

**Figure 3.**
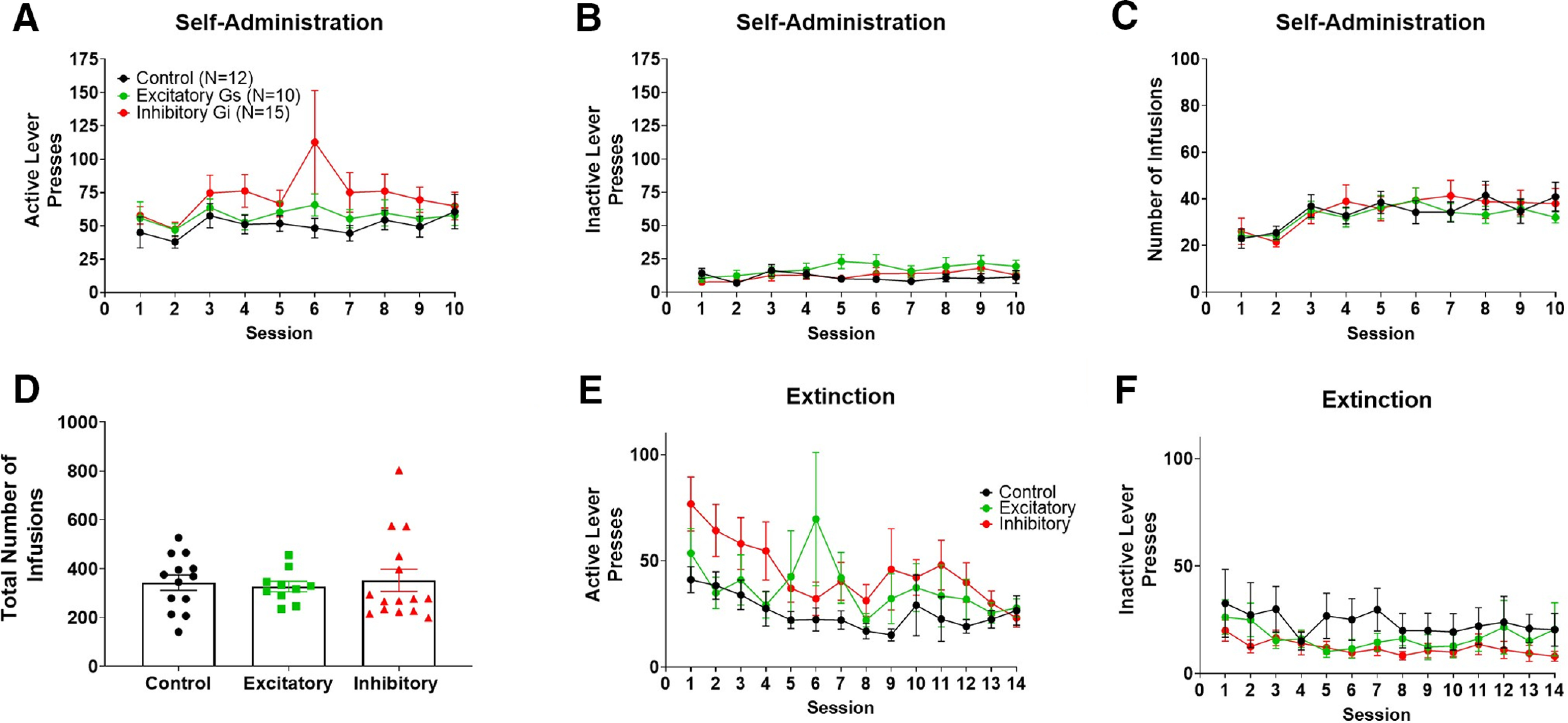
Control, excitatory, and inhibitory DREADD-expressing animals did not differ in rates of nicotine self-administration or extinction lever pressing. Regardless of the type of DREADD expression, animals showed no difference in active (***A***) or inactive (***B***) lever pressing throughout self-administration. Additionally, animals not differ in the number of infusions across self-administration sessions (***C***) or the total number of nicotine infusions earned throughout the 10 non-consecutive criteria-making sessions (***D***). Data points within ***D*** represent individual animal values. DREADD groups did not differ in active (***E***) or inactive (***F***) lever pressing throughout extinction. Control, excitatory and inhibitory DREADD-expressing rats are depicted in black, green, and red, respectively. Numbers in legend of panel ***A*** represent the number of animals in each group.

A two-way ANOVA where session and treatment (DREADD type; control: *N* = 12; excitatory: *N* = 10 and inhibitory: *N* = 15 DREADD-expressing animals) were considered factors revealed a significant effect of treatment (*F*_(2,350)_ = 8.7; *p *<* *0.001) but not session (*F*_(9,350)_ = 1.2; *p *>* *0.05) on active lever pressing. However, no interaction was observed (*p *>* *0.05). . A significant effect of treatment (*F*_(2,350)_ = 6.7; *p *<* *0.01) but not session (*F*_(9,350)_ = 1.0; *p *>* *0.05) on inactive lever pressing. However, no interaction was observed (*p *>* *0.05). A *post hoc* comparison revealed no differences between DREADD type within sessions (*p *>* *0.05). A two-way ANOVA also revealed a significant effect of session (*F*_(9,350)_ = 3.2; *p *<* *0.01) but not DREADD type (*F*_(2,350)_ = 0.6; *p* > 0.05) on nicotine infusions throughout self-administration. No interaction was observed. Additionally, a *post hoc* comparison revealed no differences between DREADD type within sessions. Lastly, control, excitatory, and inhibitory DREADD-expressing animals did not differ in total number of nicotine infusions earned across the 10-criteria making sessions (*p *>* *0.05; Fig. 3). An effect of treatment and session on active lever pressing was observed throughout extinction sessions (*F*_(2,504)_ = 12.75; *p *<* *0.01 and *F*_(13,504)_ = 2.2; *p *<* *0.01, respectively). No interaction was observed (*p* > 0.05). Additionally, an effect of treatment on inactive lever pressing was observed throughout extinction sessions (*F*_(2,504)_ = 10.5; *p *<* *0.05), yet a *post hoc* comparison revealed no differences between DREADD groups within sessions.

### ChI DREADD validation using immunohistochemistry and electrophysiology

**Figure 4.**
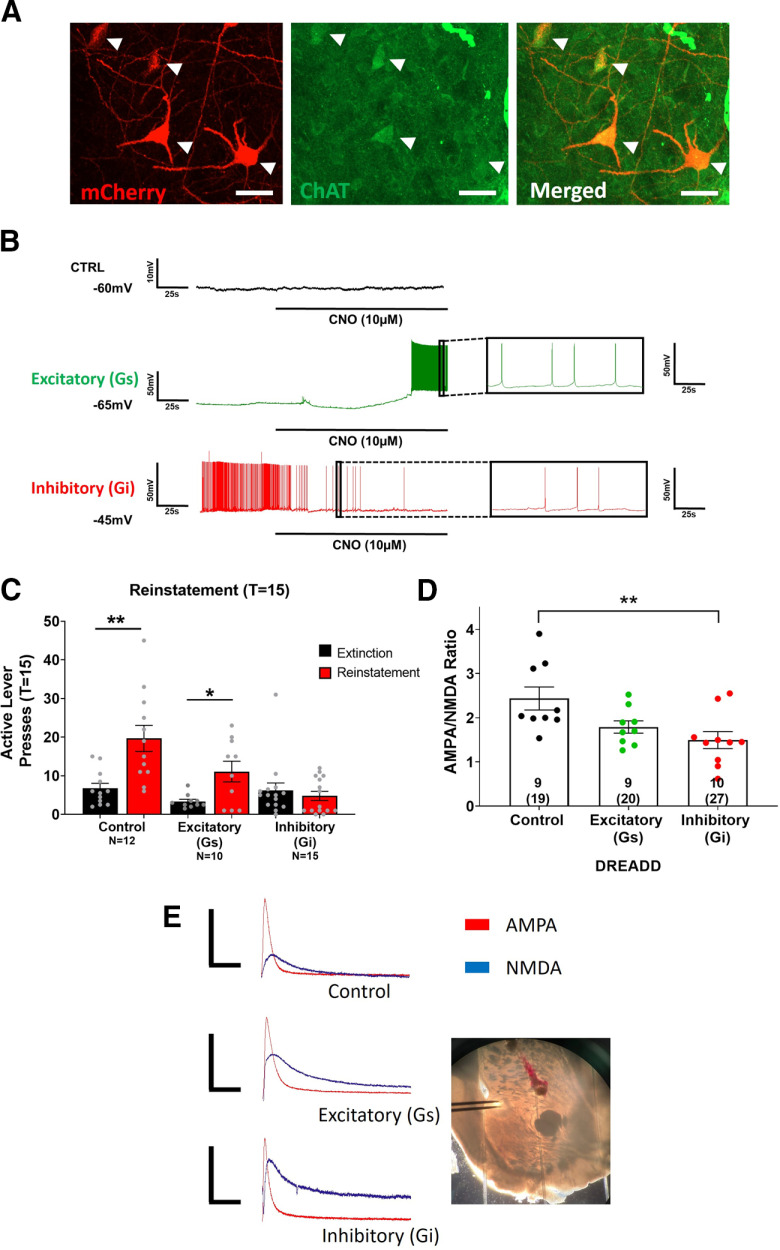
Chemogenetic ChI inhibition prevented cue-induced nicotine seeking and reduced A/N ratio. ***A***, The DREADD constructs used in the current study readily express mCherry in neurons (left panel). ChAT labeled cell bodies within the NAcore (middle panel), which co-expressed with all mCherry labeled neurons (right panel). Arrows depict cell bodies. Scale bar: 30 μm. ***B***, CNO bath application had no effect on control virus expressing ChIs (top; black), promoted firing in the excitatory DREADD-expressing ChI (middle; green); and blunted firing in the inhibitory DREADD-expressing ChI (bottom; red). ***C***, In control and excitatory DREADD-expressing animals, active lever pressing was increased during cue-induced reinstatement compared with extinction following intra-NAcore CNO treatment. In animals expressing the inhibitory DREADD, CNO inhibition of ChIs prevented cue-induced nicotine reinstatement, where active lever pressing during reinstatement was not different from extinction; *p <* *0.05 versus extinction, **p <* *0.01 versus extinction; n.s., non-significant. Inset numbers represent number of animals. ***D***, A/N ratio was reduced in animals with ChI inhibition following reinstatement [T(time) = 15] relative to control DREADD-expressing animals. The inset numbers within ***D*** represent number of animals with the total number of cells recorded from within parentheses. Representative AMPA and NMDA traces for each DREADD type is shown in panel ***E***. A picture depicting the NAcore and stimulating electrode placement is also shown. Numbers in bars represent animal number and numbers in parentheses represent the total number of cells. Data points within ***C***, ***D*** represent individual animal values. Scale bars (*x*, *y*):  50 ms, 100 pA; **p <* *0.01.

Using immunohistochemistry, all three vectors (two cre-dependent DREADDs and the cre-dependent mCherry-expressing control) were validated to ensure specific targeting of ChIs. DREADD-labeled neurons (mCherry; Fig. 4*A*, left panel) co-expressed ChAT protein (Fig. 4*A*, middle and right panel). Recordings from mCherry-tagged DREADD-labeled ChIs confirmed functionality of all DREADDs used. Bath application of CNO (10 μm) did not alter the resting membrane or spiking in control mCherry-expressing ChIs, induced firing in excitatory G_s_- DREADD-expressing ChIs and inhibited action potential firing in inhibitory G_i-_DREADD-expressing ChIs (Fig. 4*B*).

### ChI inhibition prevents cue-induced nicotine reinstatement

A two-way ANOVA with treatment (DREADD type) as between- and session (extinction vs reinstatement) as within-subject variables revealed a main effect of treatment (*F*_(2,70)_ = 8.4; *p *<* *0.01; Fig. 4*C*) and session (*F*_(2,70)_ = 13.2; *p *<* *0.01; Fig. 4*C*). Additionally, an interaction between treatment and session was observed (*F*_(2,70)_ = 6.8; *p *<* *0.01; Fig. 4*C*). Bonferroni *post hoc* multiple comparisons revealed that control CNO-treated rats increased active lever pressing during cue-induced reinstatement (T = 15) relative to the first 15 min of extinction (*t*_(70)_ = 4.4; *p *<* *0.01; Fig. 4*C*). Additionally, ChI activation with intra-NAcore administration of CNO did not prevent cue-induced reinstatement in animals expressing the excitatory DREADD, as the number of active lever presses during the cue-induced reinstatement session (T = 15) was higher than that observed during the first 15 min of extinction (*t*_(70)_ = 2.2; *p *<* *0.05; Fig. 4*C*). However, inhibition of ChIs with CNO prevented cue-induced nicotine reinstatement in inhibitory DREADD-expressing animals, where the number of active lever presses during reinstatement (T = 15) was not different from the first 15 min of extinction (*p *>* *0.05; Fig. 4*C*). No differences in inactive lever pressing were observed relative to extinction for any group (data not shown). No sex differences in active lever pressing within groups were observed (*p *>* *0.05).

### Inhibition of ChIs reduces the A/N ratio observed in MSNs

**Figure 5.**
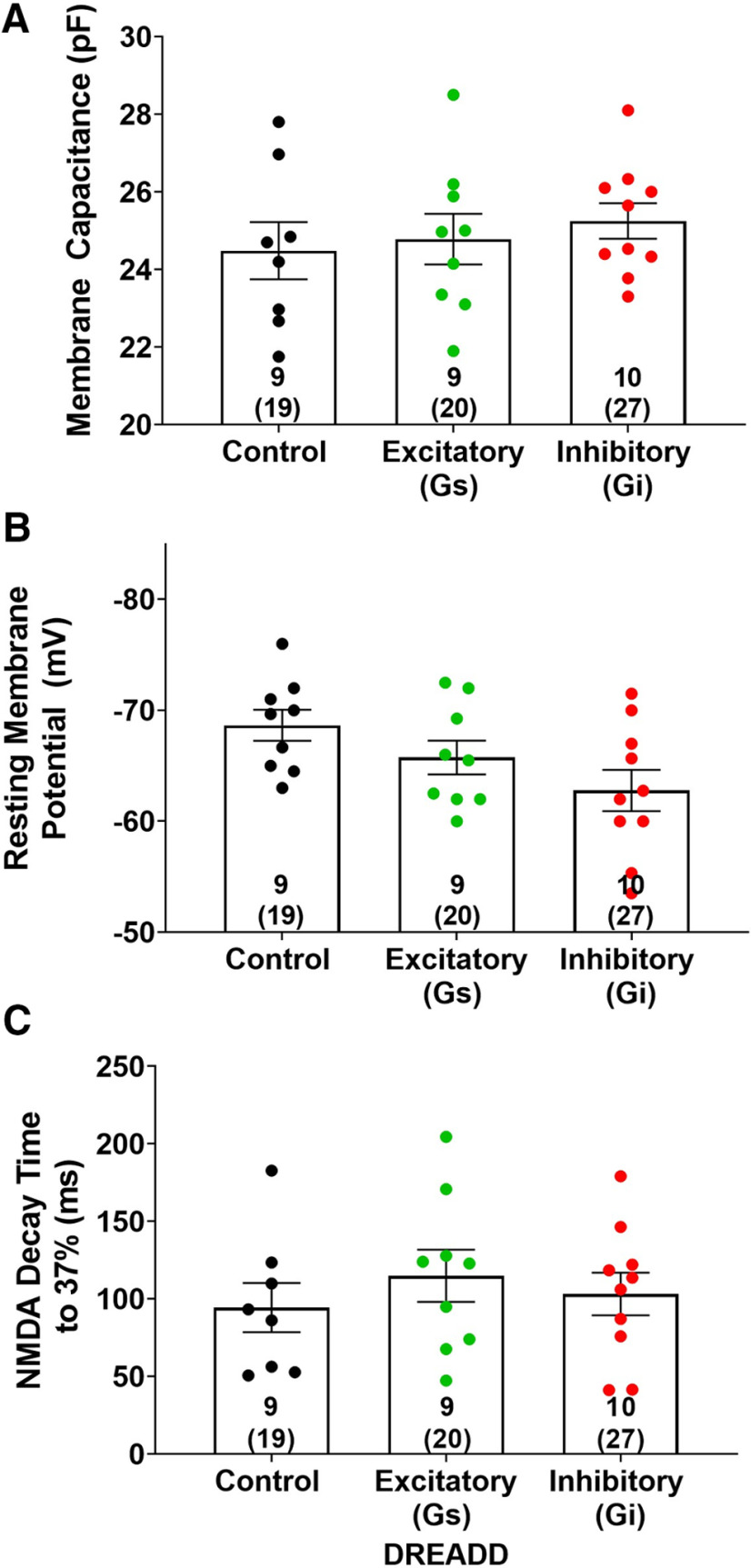
Cellular capacitance, resting membrane potential and NMDA decay time did not differ between DREADD-expressing groups. Cellular capacitance (***A***), resting membrane potential (***B***), and NMDA decay to 37% of the peak amplitude (***C***) did not differ between control, excitatory, or inhibitory DREADD-expressing animals following cue-induced reinstatement. The inset numbers represent number of animals with the total number of cells recorded from within parentheses. Data points within represent individual animal values.

Immediately following a 15-min cue-induced reinstatement session, NAcore MSN recordings were conducted from acute slices derived from animals expressing either control, inhibitory, or excitatory DREADD constructs. An ANOVA revealed a main effect of treatment (DREADD type) on A/N ratio (*F*_(2,25)_ = 5.7; *p *<* *0.01; Fig. 4*D*). *Post hoc* comparisons revealed that inhibition of ChI activity resulted in a smaller A/N relative to control animals following the 15-min cue-induced reinstatement session (*t*_(25)_ = 3.3; *p *<* *0.01; Fig. 4*D*). However, no differences were observed between rats in which ChIs were activated relative to any other group (*p *>* *0.05; Fig. 4*D*). Additionally, one-way ANOVAs revealed no differences in MSN cellular capacitance (*p *>* *0.05; Fig. 5*A*), resting membrane potential (*p *>* *0.05; Fig. 5*B*), or NMDA decay time, as measured by the time to reach 37% of peak amplitude (*p *>* *0.05; Fig. 5*C*), between control, excitatory or inhibitory DREADD-expressing animals. No sex differences in A/N or NMDA decay within groups were observed (*p *>* *0.05).

### Active lever pressing during reinstatement is positively correlated with MSN A/N ratio

**Figure 6.**
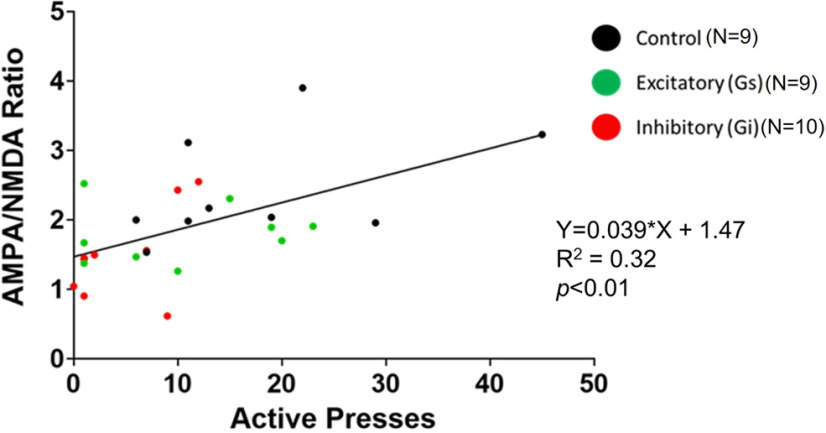
Active lever presses during reinstatement was positively correlated with MSN A/N ratio. The number of active lever presses and A/N ratio for each animal is shown. The number of animals plotted for each group is depicted as *N* within the figure. The A/N ratio measured for each cell was averaged across all cells from the same animal (between one and four cells/animal) to obtain the A/N for each animal which was then compared with the number of active lever presses during the 15-min reinstatement session. Group assignment is depicted as follows: control DREADD-expressing rats are shown as black dots, excitatory DREADD-expressing rats are shown as green, and inhibitory DREADD-expressing rats are shown as red.

The number of active lever presses and A/N were plotted for each animal. The A/N ratio measured for each cell was averaged across all cells from the same animal (between one and four cells/animal) to obtain the A/N for each animal. A linear regression analysis revealed a relationship between the number of active lever presses during reinstatement (T = 15) and the A/N ratio (*F*_(1,26)_ = 12.4; *p *<* *0.01; Fig. 6). No sex differences in the slope were observed (*p *>* *0.05; data not shown).

## Discussion

The present results show that chemogenetic inhibition of NAcore ChIs inhibited cue-induced nicotine-seeking behavior. Additionally, inhibition of ChIs reduced A/N relative to control animals receiving the same dose of CNO. Further, the magnitude of reinstated nicotine seeking was positively correlated with the A/N ratio, where higher active lever pressing was associated with a larger A/N ratio, an effect similar to those observed in other studies (Gipson et al., 2013a). Together, these results demonstrate that accumbens ChIs exert control over cue-induced nicotine-seeking behavior and MSN synaptic physiology within the NAcore (Marchi et al., 2002).

### Interactions between ChIs, ACh, nAChRs, and drug-motivated behavior

The cholinergic system has been heavily implicated in reward-related behaviors. Interestingly, elevated levels of ACh have been observed in the nucleus accumbens following cocaine (Crespo et al., 2006; Yee et al., 2011), remifentanil (Crespo et al., 2006), nicotine (Rada et al., 2001; Mansvelder et al., 2003), and alcohol (Larsson et al., 2005). Further, elevated levels of ACh in the accumbens parallel the reinforcing effects of both intravenous cocaine and remifentanil (Crespo et al., 2006), providing evidence for ACh mediation of drug-motivated behavior. Interestingly, ACh release in the accumbens is also enhanced during contingent versus non-contingent intravenous cocaine administration, suggesting that ACh release may be differentially mediated by volitional versus passive drug exposure (Mark et al., 1999). ChIs are also heavily implicated in stimulus-response associations (Aosaki et al., 1994; Suzuki et al., 2001; Joshua et al., 2008; Collins et al., 2019), thus it is not surprising that silencing accumbens ChIs prevents cocaine conditioned reward (Witten et al., 2010). Witten and colleagues suggest that acute silencing of accumbens ChIs disrupts drug-related learning without affecting conditioning (Witten et al., 2010). Thus, while ACh release is primarily driven by activation of ChIs within the accumbens, acute silencing of ChIs in this region can disrupt drug-related learning. ACh-releasing ChIs have also been shown to exert powerful modulatory control over MSNs and dopaminergic tone within the NAcore (Cachope et al., 2012; Yorgason et al., 2017). Within the current study, we report that inhibition of ChIs prevented cue-induced nicotine reinstatement, further suggesting that inhibiting ACh release within the NAcore may prevent nicotine seeking in part by blunting the stimulus-response association.

nAChRs are densely expressed on ChIs as well as glutamatergic and dopaminergic terminals throughout the NAcore (Zhou et al., 2001; Zhang and Sulzer, 2004; Zappettini et al., 2014; Girasole and Nelson, 2015), allowing for extensive control of accumbens circuitry. Further, nAChRs have been heavily implicated in the molecular aspects underlying addiction. For example, nAChRs are upregulated within the accumbens following voluntary ethanol consumption (Larsson et al., 2005). Moreover, nAChR inhibition also reduces cocaine place preference and cocaine sensitivity (Zachariou et al., 2001) and nAChR antagonism within the VTA attenuates cue-induced cocaine seeking (Nunes et al., 2019). While the role of nAChRs have been explored across multiple drugs of abuse, their involvement in nicotine use and addiction has been most prominently studied. Nicotine directly activates and leads to prolonged desensitization of nAChRs, which contributes to the reinforcing properties of nicotine (Picciotto et al., 1998, 2008; Mansvelder and McGehee, 2002; Mansvelder et al., 2003; Giniatullin et al., 2005). Multiple studies have shown that antagonism of nAChRs reduces active lever pressing in animals self-administering intravenous nicotine (Watkins et al., 1999; Markou and Paterson, 2001; Toll et al., 2012; Liu, 2014), although nAChR modulation specifically within the NAcore in a cue-induced nicotine reinstatement paradigm following self-administration has yet to be studied. Regardless, the findings of these highlighted prior studies support the hypothesis that the NAcore cholinergic system plays a critical role in nicotine addiction.

### Interactions between ChIs, dopamine release, and MSN synaptic plasticity

While the main input of MSNs is glutamatergic innervation from the cortex, ChIs also receive excitatory cortical inputs. Interestingly, cortical stimulation evokes excitatory responses in ChIs before MSNs, as depicted by postsynaptic current latencies and rise times. Specifically, the postsynaptic current latency was slower in MSNs relative to ChIs, indicating that ChIs are excited by excitatory inputs before MSNs (Fino et al., 2008). With MSNs being downstream targets of ChIs, cortical inputs thus have the ability to directly and indirectly modulate the excitability and membrane potential of MSNs through direct synaptic connectivity and ChIs, respectively. Given these findings, ChIs may have the ability to alter MSN synaptic plasticity even before MSNs receiving cortical input. As such, alterations in MSN synaptic plasticity may be because of modifications in the timing and order of the presynaptic and postsynaptic activity at cortico-striatal synapses. In addition to ChIs ability to directly alter MSN synaptic excitability, ChIs modulate dopamine release within the accumbens by activating nAChRs located on dopaminergic terminals (Nelson et al., 2014). Specifically, synchronous activity of the ChI population can induce DA release by bypassing action potential generation within the dopaminergic soma (Cachope et al., 2012; Threlfell et al., 2012). Not surprisingly, cortical glutamatergic inputs targeting the accumbens can modify dopamine release indirectly through activation of ionotropic glutamate receptors located on ChIs (Kosillo et al., 2016). MSNs are particularly susceptible to dopaminergic modulation, where they are distinguished into two major subtypes, classified by their expression of either D1-type or D2-type dopamine receptors. Given their extensive expression of dopamine receptors, dopamine has the ability to extensively modify both the structure and synaptic function of striatal circuits. Current hypotheses suggest that dopaminergic neurons innervating the striatum modify the strength of corticostriatal synapses of MSNs, contributing to action selection behaviors (Yin and Knowlton, 2006; Cohen and Frank, 2009) such as drug seeking.

In the current study, we report that inhibition of ChIs reduced the A/N ratio of MSNs relative to control, where ChI activity was unaltered. Given the complexity of this circuitry, it is difficult to pinpoint the exact mechanism driving the observed changes in A/N ratio of MSNs in the current study. However, the reduction in ChI activity because of chemogenetic inhibition may prevent pertinent crosstalk between glutamatergic inputs, ChIs, dopamine release, and MSNs, resulting in a reduction of A/N ratios in MSNs. Additionally, here we report that activation of ChIs reduced the A/N ratio, consistent with a recent study showing that ChI activation during the extinction phase of cocaine CPP reduced the A/N ratio of MSNs, although not relative to controls (Lee et al., 2016). Furthermore, the same study reported a reduction in miniature EPSPs in MSNs as a result of ChI activation, suggesting a reduction in MSN excitatory tone (Lee et al., 2016). However, it may also be possible that chemogenetic activation of ChIs did not promote reinstatement beyond control virus-expressing animals in the current study because of ChI autoregulation, where ChI overexcitation and enhanced ACh release induced by CNO may lead to activation of muscarinic M4 autoreceptors located on ChIs. Activation of these receptors induces membrane hyperpolarization and inhibition of ChI calcium channels (Girasole and Nelson, 2015), allowing for self-regulation and a blunting of further ACh release. Because of overexcitation of ChI activity caused by CNO, autoregulatory mechanisms may have prevented ChIs from promoting glutamate release and altering MSN synaptic plasticity. However, because these mechanisms were not examined within the current manuscript, future studies testing these mechanisms are warranted.

### Limitations of the current study

While the current study used chemogenetics to explore the effects of ChIs on nicotine-seeking and MSN synaptic plasticity, the effects of ChIs on other components within the accumbens circuitry including the glutamatergic inputs from the cortex as well as dopamine terminals were not explored. As discussed above, the synaptic connectivity of MSNs has been shown to be altered by ChIs, dopamine release, as well as cortical inputs. Thus, to fully characterize the role of ChIs in mediating nicotine seeking and accumbens plasticity, additional studies addressing the interactions of ChI-dopamine-MSN interactions as well as MSN subtypes are warranted. These additional studies would further uncover key components contributing to MSN synaptic modifications within the NAcore observed in the current study as well as the regulatory role of the cholinergic system in mediating nicotine-seeking behavior.

Recent studies have demonstrated that ChAT::cre rats display elevated levels of the vesicular acetylcholine transporter protein, as well as altered motor, anxiety and attentional task performance (Mantanona et al., 2019). While it is important to note that these effects could affect the conclusions drawn within the current study, we have recently compared the self-administration characteristics of wild-type Long–Evans rats with ChAT::cre-positive Long–Evans rats and found no differences in total infusions across self-administration sessions or active and inactive lever pressing (Leyrer-Jackson et al., 2020). Thus, while others have reported behavioral differences of ChAT::cre rats compared with wild type (Mantanona et al., 2019), we believe that the lack of differences in self-administration characteristics demonstrate that these groups do not differ in their nicotine-seeking behaviors. However, future studies would benefit from comparing the MSN synaptic physiology and accumbens circuitry between ChAT::cre and wild-type rats.

In conclusion, we report that ChI inhibition prevents cue-induced nicotine reinstatement and blunts MSN A/N. These results suggest that the cholinergic system heavily modulates nicotine-seeking behavior and associated glutamate plasticity. Thus, ChIs may be an essential modulator of MSN synaptic plasticity, which may play a role in nicotine seeking, that has been previously overlooked. Taken together, the current findings illustrate an additional component of the highly complex neurophysiological underpinnings of nicotine relapse, where ChIs mediate control of MSN A/N and thus play a pertinent role in transitioning nicotine craving to seeking.

## Synthesis

Reviewing Editor: Christie Fowler, University of California, Irvine Decisions are customarily a result of the Reviewing Editor and the peer reviewers coming together and discussing their recommendations until a consensus is reached. When revisions are invited, a fact-based synthesis statement explaining their decision and outlining what is needed to prepare a revision will be listed below. The following reviewer(s) agreed to reveal their identity: Nii Addy, Ryan Drenan.

This manuscript provides a new understanding of the nucleus accumbens glutamatergic plasticity mechanisms by which nucleus accumbens cholinergic interneurons mediate cue-induced nicotine-seeking behavior. This represents an important issue regarding the mechanisms underlying nicotine relapse-related behavior. For the revised manuscript, while the requested control study examining t-SP would be been preferred, the reviewers recognize the limitations imposed with the pandemic restrictions. Given this, the reviewers were asked to assess whether the current findings presented provide a scientific advance with the removed data sets, and all agreed that the current version of the manuscript represents an advance for the field. Thus, the authors are to be commended for these changes. However, it was also noted that there are still some outstanding concerns, which need to be addressed in a revised submission. Of highest importance, a statistical table needs to be included that provides the name of the test performed, value of the test statistic (t, F, etc.), the degrees of freedom, exact p value, repeated measures analysis (if applicable), statistical corrections for multiple comparisons (if applicable), and avoid using the term “significant” or “non-significant. Finally, please also include data points on all bar graphs.

Other points to address:

P2 Line 34: “...within the nucleus accumbens MSNs...” the authors only examined the NAcore.

P4 Line 73: “...(nAChRs), which are expressed on cholinergic interneurons (ChIs) in striatum.” The paper cited to support this statement is a review, and that review cited another review to make a similar assertion. In fact, there is little to no evidence for expression of nAChRs in cholinergic interneurons. There may be scant traces here and there in the literature, but no replicable findings can support this statement. Please remove it.

P4 Line 81-83 “ACh release within the NAcore....” the wording within this sentence regarding Ach release and cocaine/remifentanil is confusing.

P 8, Line 191: A statement about the injections being made bilaterally should be included (as in the methods it states that guide cannula were placed bilaterally)

P 12 Line 285: The sentence beginning with “Because active lever....” is a sentence fragment and should be rewritten, along with the sentences that follow. Also, please justify the assertion that active reinforced versus active non-reinforced lever responses are governed by different mechanisms.

P 12 Line 290: State “...post-hoc didn’t show differences across session..” but do not state whether there was an interaction. As stated above, include the statistical results regardless of whether there is an interaction.

P13 Line 294/295: why are they switching apparently between statistical techniques (ANOVA, and linear regression)?

P13: Paragraph about sex differences: The whole paragraph should be split into two as it is looking at two different things (sex and DREADD type).

Figure 1B: A dashed line would be helpful to delineate between the self-administration and extinction trials

Figure 1C is unnecessary; doesn’t add any additional information, especially because this difference in the types of active lever responses (Reinforced vs TO) isn’t discussed within the extinction paradigm or within regards to sex differences.

Figure 2: The authors reveal that they published a paper earlier this year (Leyrer-Jackson et al, Exp. Clin. Neuropsychopharm. 2020) that shows a sex difference in the LE strain with regard to nicotine SA. Please explain the discrepancy, therefore, between those published results and the apparent lack of a sex difference as shown in Figure 2. Further, the authors state that the data from the present submission was taken from the larger study that was published earlier this year. That seems to suggest that the data subset represented here was not representative of the larger study.

Figure 4C: Are the authors examining total active lever responses (i.e. those that lead to a reward, and any made during the time-out period)? Additionally, it appears there may have an effect over the first 15-minutes between the control and the excitatory group during extinction? And if there was, how does this reflect the findings discussed in Figure 3?

The authors present some of the sex differences (i.e. Figure 2), but not data from reinstatement. Is there a reason for that?

Figure 4D: I am a bit confused about what the inset number represents as there are two values.

Figure 6: Is the sample size regarding the behavioral data (active presses) the same as the number of animals used in the MSN A/N ratio analysis?

P17 second paragraph: The authors discuss that within the core, there is powerful modulatory control but in the next sentence talk about the elevation of ACh in the nucleus accumbens. If the authors are focused on discussing the core, then the supporting arguments should be relative to this structure.

P18 Line 427/428: Sentence needs to be reworded-confusing.

P18 Line 438 “antagonism...VTA” unnecessary. Also as the authors highlight in the following sentence, a discussion of nAChRs within the NAcore is warranted.

P23 Line 541 “...we hypothesize that activation didn’t promote reinstatement...” I don’t believe this statement reflects the findings from Figure 4.

## Author Response

Synthesis Statement for Author (Required):

This manuscript provides a new understanding of the nucleus accumbens glutamatergic plasticity mechanisms by which nucleus accumbens cholinergic interneurons mediate cue-induced nicotine-seeking behavior. This represents an important issue regarding the mechanisms underlying nicotine relapse-related behavior. For the revised manuscript, while the requested control study examining t-SP would be been preferred, the reviewers recognize the limitations imposed with the pandemic restrictions. Given this, the reviewers were asked to assess whether the current findings presented provide a scientific advance with the removed data sets, and all agreed that the current version of the manuscript represents an advance for the field. Thus, the authors are to be commended for these changes.

We sincerely appreciate the consideration of limitations due to the pandemic, and greatly appreciate the positive review by reviewers. We have noted the concerns outlined below, responded to each in italics, and have revised the manuscript accordingly.

However, it was also noted that there are still some outstanding concerns, which need to be addressed in a revised submission. Of highest importance, a statistical table needs to be included that provides the name of the test performed, value of the test statistic (t, F, etc.), the degrees of freedom, exact p value, repeated measures analysis (if applicable), and statistical corrections for multiple comparisons (if applicable).

A table has now been included within the revised manuscript as Table 1.

In the manuscript, please avoid using the term “significant” or “non-significant. Finally, please also include data points on all bar graphs.

These terms have now been omitted, and data points have now been added to all bar graphs.

Other points to address:

P2 Line 34: “...within the nucleus accumbens MSNs...” the authors only examined the NAcore.

This has been fixed.

P4 Line 73: “...(nAChRs), which are expressed on cholinergic interneurons (ChIs) in striatum.” The paper cited to support this statement is a review, and that review cited another review to make a similar assertion. In fact, there is little to no evidence for expression of nAChRs in cholinergic interneurons. There may be scant traces here and there in the literature, but no replicable findings can support this statement. Please remove it.

This has been removed.

P4 Line 81-83 “ACh release within the NAcore....” the wording within this sentence regarding Ach release and cocaine/remifentanil is confusing.

Wording has now been revised for clarity.

P 8, Line 191: A statement about the injections being made bilaterally should be included (as in the methods it states that guide cannula were placed bilaterally)

This statement has been included.

P 12 Line 285: The sentence beginning with “Because active lever....” is a sentence fragment and should be rewritten, along with the sentences that follow. Also, please justify the assertion that active reinforced versus active non-reinforced lever responses are governed by different mechanisms.

This sentence and those that follow have now been edited for flow. To the point of separating these two types of active lever presses, we provide the following justification: Active lever presses that result in a reinforcer are distinct from those that do not. In this case, we are distinguishing between lever presses that result in the delivery of the infusion versus those that are made during the infusion itself (time-out responses). Early studies in the experimental analysis of behavior noted that operant extinction, which in this case is akin to responses made during the time-out, can be defined in two ways: either by the omission of a previously delivered reinforcer, or as the absence of a contingency between response and reinforcer. Given that the reinforcer (the infusion in this case) is not contingently presented following emission of a time-out active lever press, the latter definition applies here. Thus, active lever presses made during an infusion (which do not directly result in the infusion itself, or prolong the duration of the infusion) are distinct from those that result in the temporally contiguous delivery of the reinforcer (in this case, nicotine). For references on this usage, please see Lattal and Lattal, 2012; Rescorla and Skucy, 1969. This justification and these references have now been included in the manuscript within the methods section.

P 12 Line 290: State “...post-hoc didn’t show differences across session..” but do not state whether there was an interaction. As stated above, include the statistical results regardless of whether there is an interaction.

This has now been included.

P13 Line 294/295: why are they switching apparently between statistical techniques (ANOVA, and linear regression)?

We have repeated utilized linear regression to show acquisition of drug self-administration (e.g., Gipson et al., 2011, Psychopharmacology; Leyrer-Jackson et al. 2020, Brain Structure and Function; Leyrer-Jackson et al. 2020 Ex. Clin. Psychopharm. Powell et al. 2019, Physiological Reports). However, we have removed this analysis for exploring changes in acquisition within the manuscript for consistency. However, we have kept the linear analysis for exploring the relationship between A/N and active lever pressing during reinstatement.

P13: Paragraph about sex differences: The whole paragraph should be split into two as it is looking at two different things (sex and DREADD type).

This paragraph has now been split into two paragraphs, one on sex and one on DREADD type.

Figure 1B: A dashed line would be helpful to delineate between the self-administration and extinction trials

A dashed line has now been included.

Figure 1C is unnecessary; doesn’t add any additional information, especially because this difference in the types of active lever responses (Reinforced vs TO) isn’t discussed within the extinction paradigm or within regards to sex differences.

Figure 1C has now been omitted.

Figure 2: The authors reveal that they published a paper earlier this year (Leyrer-Jackson et al, Exp. Clin. Neuropsychopharm. 2020) that shows a sex difference in the LE strain with regard to nicotine SA. Please explain the discrepancy, therefore, between those published results and the apparent lack of a sex difference as shown in Figure 2. Further, the authors state that the data from the present submission was taken from the larger study that was published earlier this year. That seems to suggest that the data subset represented here was not representative of the larger study.

We appreciate this concern and realize that it was ambiguous within the previous version of the manuscript. Our previously published paper (Leyrer-Jackson et al., Exp. Clin. Psychopharm 2020) explored sex and strain differences between Long Evans and Sprague Dawley rats. In the previously published paper, we report sex differences within the Sprague Dawley groups, where SD females had less total nicotine infusions across self-administration sessions than SD males. Additionally, we reported that LE females infused more nicotine across the self-administration sessions than SD females (an effect of strain). However, similar to results within the current study, we observed no differences in total nicotine infusions between LE males and females - nor did we observe differences in infusions across sessions between the two groups. In the revised manuscript, we have now explicitly stated that the results reported here are in line with those of the recently published manuscript.

Figure 4C: Are the authors examining total active lever responses (i.e. those that lead to a reward, and any made during the time-out period)? Additionally, it appears there may have an effect over the first 15-minutes between the control and the excitatory group during extinction? And if there was, how does this reflect the findings discussed in Figure 3?

Figure 4C represents total number of active lever presses during the 15 minute cue induced reinstatement session. Because no infusions were delivered during the reinstatement sessions, they are not separated by infusion vs. TO responses. There was no effect between the control and excitatory group during the first 15 minutes of the extinction period thus, they accurately correlate with the results shown in figure 3.

The authors present some of the sex differences (i.e. Figure 2), but not data from reinstatement. Is there a reason for that?

Within the original manuscript, we discussed sex differences for each of the findings - in general, these are stated as the last sentences within each results section. We have not displayed them in figures because all of these comparisons were non-significant.

Figure 4D: I am a bit confused about what the inset number represents as there are two values.

The inset numbers represent the number of animals and the number of cells recorded from are within parentheses. This information has now been included within the figure legends.

Figure 6: Is the sample size regarding the behavioral data (active presses) the same as the number of animals used in the MSN A/N ratio analysis?

We thank the reviewers for catching this error in reported N within this figure - This has been updated to accurately reflect the N, which is the number of animals. The A/N ratio of all cells for each animal were averaged to give an animal average, which was compared to their behavior. This information has also been included within the figure legend in addition to the results section.

P17 second paragraph: The authors discuss that within the core, there is powerful modulatory control but in the next sentence talk about the elevation of ACh in the nucleus accumbens. If the authors are focused on discussing the core, then the supporting arguments should be relative to this structure.

We have re-arranged this paragraph to first discuss the accumbens followed by the accumbens core. We believe the restructuring provides proper flow from general to more specific.

P18 Line 427/428: Sentence needs to be reworded-confusing.

This sentence has been modified

P18 Line 438 “antagonism...VTA” unnecessary. Also as the authors highlight in the following sentence, a discussion of nAChRs within the NAcore is warranted.

We have removed reference to antagonsim of nAChRs within the VTA, and have now highlighted the lack of studies examining NAcore-specific nAChR modulation of nicotine seeking behaviors following self-administration. To our knowledge, nAChR manipulation of the NAcore specifically has yet to be conducted.

P23 Line 541 “...we hypothesize that activation didn’t promote reinstatement...” I don’t believe this statement reflects the findings from Figure 4.

We have now added a statement to this sentence “...did not promote reinstatement beyond control virus expressing animals may be due to...” - while excitatory expressing animals did reinstate to nicotine paired cues, they did not reinstate as robustly as control virus expressing animals. We believe the addition to this sentence removes ambiguity and accurately reflects the hypothesis stated
